# Diagnostic delay of associated interstitial lung disease increases mortality in rheumatoid arthritis

**DOI:** 10.1038/s41598-021-88734-2

**Published:** 2021-04-28

**Authors:** Esteban Cano-Jiménez, Tomás Vázquez Rodríguez, Irene Martín-Robles, Diego Castillo Villegas, Javier Juan García, Elena Bollo de Miguel, Alejandro Robles-Pérez, Marta Ferrer Galván, Cecilia Mouronte Roibas, Susana Herrera Lara, Guadalupe Bermudo, Marta García Moyano, Jose Antonio Rodríguez Portal, Jacobo Sellarés Torres, Javier Narváez, María Molina-Molina

**Affiliations:** 1 Hospital Universitario Lucus Augusti, Rúa Dr. Ulises Romero, 1, 27003 Lugo, Spain; 2 Hospital de La Santa Creu I Sant Pau, Barcelona, Spain; 3 Complejo Asistencial Universitario de León, León, Spain; 4 Hospital Universitari de Bellvitge-IDIBELL, L’Hospitalet del Llobregat, Barcelona, Spain; 5 Hospital Universitario Virgen Macarena, Sevilla, Spain; 6 Complejo Hospitalario Universitario de Vigo, Pontevedra, Spain; 7 Hospital Universitario Dr. Peset, Valencia, Spain; 8 Hospital Universitario de Cruces, Barakaldo, Vizcaya Spain; 9 Hospital Universitario Virgen del Rocío, Sevilla, Spain; 10 Hospital Clínic, Barcelona, Spain

**Keywords:** Prognostic markers, Rheumatoid arthritis

## Abstract

Rheumatoid arthritis (RA) is a systemic autoimmune disease whose main extra-articular organ affected is the lung, sometimes in the form of diffuse interstitial lung disease (ILD) and conditions the prognosis. A multicenter, observational, descriptive and cross-sectional study of consecutive patients diagnosed with RA-ILD. Demographic, analytical, respiratory functional and evolution characteristics were analyzed to evaluate the predictors of progression and mortality. 106 patients were included. The multivariate analysis showed that the diagnostic delay was an independent predictor of mortality (HR 1.11, CI 1.01–1.23, *p* = 0.035). Also, age (HR 1.33, 95% CI 1.09–1.62, *p* = 0.0045), DLCO (%) (HR 0.85, 95% CI 0.73–0.98, *p* = 0.0246), and final SatO2 (%) in the 6MWT (HR 0.62, 95% CI 0.39–0.99, *p* = 0.0465) were independent predictor variables of mortality, as well as GAP index (HR 4.65, 95% CI 1.59–13.54, *p* = 0.0051) and CPI index (HR 1.12, 95% CI 1.03–1.22, *p* = 0.0092). The withdrawal of MTX or LFN after ILD diagnosis was associated with disease progression in the COX analysis (HR 2.18, 95% CI 1.14–4.18, *p* = 0.019). This is the first study that highlights the diagnostic delay in RA-ILD is associated with an increased mortality just like happens in IPF.

## Introduction

Rheumatoid arthritis (RA) is characterized by inflammation of synovial tissues and joint destruction that may involve other organs, including the lung^1^. Interstitial lung disease (ILD) is a common manifestation of RA and may precede the joint inflammation^2^. ILD associated to RA (RA-ILD) presents clinical and radiological features similar to idiopathic ILDs^3^. The prevalence of RA-ILD varies depending on the method used to diagnose the disease and the population under study^4^. Several studies based on reference cohorts have estimated a prevalence ranging between 1 and 58%^5,6^. According to Bongartz et al. approximately 1 in 10 patients with RA will be diagnosed of ILD during the course of their disease^6^.

ILD is one of the main causes of mortality in RA^4^. Predicting the evolution and outcome of RA-ILD patients is difficult because the evidence is based on small population and short-term follow-up studies. The most frequent radiological and histological pattern in RA-ILD is usual interstitial pneumonia (UIP)^7^. Furthermore, this pattern is associated with higher mortality compared to others such as non-specific interstitial pneumonia (NSIP). In fact, the survival of RA-ILD patients with a UIP pattern is closer to that patients diagnosed of idiopathic pulmonary fibrosis (IPF)^8^.

Oher factors associated with a higher mortality in RA-ILD are age, male sex, worse pulmonary function at diagnosis, duration of RA, disease RA activity, extensive lung involvement on chest HRCT and elevated serum levels of Krebs von den Lungen 6 (KL-6) biomarker^9–13^. Although these findings are all based on small series of cases, they have been reproduced consistently in different patient cohorts. Furthermore, predictive factors of disease progression are the presence of UIP pattern, high titers of anti-citrullinated cyclic peptide (ACPA) antibodies, baseline deterioration of DLCO, a %FVC decline ≥ 10% during the follow-up, and elevated serum levels of interleukin 6 (IL-6) and KL-6. However, there is no clear definition of progression specifically for RA-ILD^6,14–17^.

Other reasons that make difficult to predict survival of RA-ILD patients is the heterogeneity in their evolution and the absence of an individual variable that is sufficiently accurate to predict mortality. In this regard, the use of multidimensional risk scales can be useful for physicians. The main ones are the GAP index that combines age, sex and pulmonary physiologic values and the composite physiologic index (CPI) that only includes pulmonary physiologic characteristics^18,19^. These models were originally developed for IPF but recent studies have assessed the utility of these predictive models in RA-ILD^20^. A better predictive prognostic model could help in setting the appropriate timing for lung transplantation or optimizing treatments.

Finally, the potential harmful effect of some common medications for RA remains controversial. There is not enough evidence to recommend a specific treatment for RA-ILD, consequently the optimal treatment for RA-ILD has not yet been established^21^. Current treatment regimens usually include corticosteroid therapy, methotrexate -MTX- , leflunomide -LEF-, and biologic disease-modifying anti-rheumatic drugs (e.g. rituximab, abatacept), immunosuppressant agents (cyclophosphamide and Mycophenolate Mofetil), and antifibrotic treatment (nintedanib)^22^. In counterpoint frequently used treatments, as MTX and LEF, have also been implicated in the development of pneumonitis or exacerbation of an existing ILD^23^, although the use of MTX has been recently associated with a better outcome^24,25^. In addition, some studies have shown an increased risk of infections associated to the immunosuppressive treatments^26^.

The aims of this study are: (a) to evaluate the predictive factors of mortality, (b) to investigate the potential associated factors to ILD progression.

## Methods

### Patient population

This is a multicenter, observational, descriptive and cross-sectional study of consecutive patients with diagnosis of RA-ILD from nine Spanish hospitals. Clinical records were reviewed between 2013 and 2018. The diagnosis of RA was done fulfilling ACR/EULAR 2010 criteria^27^ and the CTD-ILD classification according to the 2003 Respiratory Spanish Society (SEPAR) consensus^3^. The ILD pattern was identified and defined by HRCT. Patients with diagnosis of different CTD or other respiratory diseases were excluded. Only patients with ≥ 3 pulmonary function test measurements during the follow-up were included.

The Regional Research Ethics Committee of Galicia approved the study protocol (Registration Code 2016/176). Each participating hospital obtained the ethic approval from the local Human Research Ethics Committee. Informed consent was obtained from each patient.

Procedures were in accordance with guidelines established in the Declaration of Helsinki, and with the principles of Good Clinical Practices (GCPs).

### Collected data and definitions

Demographic, epidemiological, radiological and treatment data were collected. Pulmonary disease progression was considered if the patient presented any of the following criteria: forced vital capacity (FVC) decrease ≥ 10%, diffusion capacity for carbon monoxide (DLCO) decrease ≥ 15%, radiological increase of lung fibrotic signs or death. Respiratory infectious events were defined as episodes of respiratory deterioration with or without respiratory failure that requires antibiotic treatment and may have required hospitalization. Heart failure or other non-respiratory reasons of hospitalization were excluded.

Two independent ILD expert radiologists evaluated the HRCT images over time and considered worsening of fibrotic radiological signs when an increase of more than 5% was identified.

Time to diagnosis of ILD from the onset of symptoms was classified as follows;  < 12 months, 12–24 months, > 24 months (the last two groups were considered diagnostic delay).

The GAP index was calculated using the continuous predictor variables sex, age, and the pulmonary functional values, through the online model (www.acponline.org/journals/annals/extras/gap)^18^. Composite physiologic index (CPI) was calculated using the formula: CPI = 91-(0.65xDLCO% pred.)–(0.53xFVC% pred.) + (0.34 × FEV1% pred.)^19^.

### Statistical analysis

The distribution of the continuous variables was verified with the Shapiro–Wilk test. The Student *T*-test was used to compare continuous variables if there was a normal distribution. If it did not have a normal distribution the Mann–Whitney U-test was used. The chi-squared test or Fisher test was used for comparison of categorical variables. Logistic regression analysis was used to identify significant variables capable of predicting respiratory infectious events.

Survival and progression differences between groups were evaluated by Kaplan–Meier analysis. Survival curves were compared with the log-rank test. A univariate Cox regression was performed to calculate the hazard ratio (HR) with a 95% confidence interval (CI) of the independent variables. A multivariate Cox regression model was carried out adjusting for all confounding variables that in univariate analysis had a *p* value < 0.2. Time to death was obtained from medical records and the censoring time was defined as the last medical visit or the end of the study at December 31, 2018.

It was considered appropriate to determine a cut-off level of CRP, ESR and RF by maximizing the Youden index from ROC curves for analysis of progression instead of using continuous data because they did not satisfy the assumption that the log hazard increased linearly with the covariate^28^. Since including variables with substantial missing data can introduce significant bias, anti-CCP antibodies and DAS28 score, which had a substantial number of missing values, were not included in the models of survival and progression.

The results were reported as mean ± standard deviation (SD). *P* < 0.05 was considered statistically significant. MedCalc Statistical Software version 14.8.1 (MedCalc Software bvba, Ostend, Belgium) was used to perform all the analysis.

## Results

### Population

A total of 106 patients with RA-ILD were included, 61 women (57.5%). The mean age was 70.21 ± 9.8 years. RA was diagnosed 126.66 ± 117.28 months before the diagnosis of ILD. In 10 patients (9.43%) the ILD was present at the onset of disease. 40.6% of patients (n = 43) were never smokers. A confident UIP pattern in the HRCT was present in 51.9% of the patients. At the moment of ILD diagnosis 10 patients (9.43%) presented severe oxygen desaturation on effort (defined as SatO2 < 88% in the 6MWT). Therefore, a delay in the ILD identification was observed in a high proportion of patients. An abnormally high levels of anti-CCP was observed in 63 cases (66.8%). The rest of the characteristics are described in Table 1.

**Table 1:** Characteristics of the entire RA-ILD cohort.

	n = 106	%	SD	Min–Max
Gender (male/female)	45/61	42.5/57.5		
Age (years)	70.21		9.8	42–89
**Tobacco**				
Current	9	8.9		
Non	43	40.6		
ormer	49	48.5		
Packs-year	48.58		49.91	10–285
RA evolution (months)	126.66		117.28	0–480
ILD evolution (months)	42.21		40.1	2–240
Respiratory symptoms duration until ILD diagnosis (months)	14.36		15.3	0–96
Cough	48	45,3		
Crackles	59	56.2		
**Dyspnea**				
mMRC 0	30	28.3		
mMRC 1	49	46.2		
mMRC 2	20	18.9		
mMRC 3	5	4.7		
**HRCT pattern**				
UIP	55	51.9		
NSIP	48	45.3		
OP	3	2.8		
ESR (mm/H)	39.39		42.54	3–367
CRP (mg/L)	25.52		58.9	0–507
RF (titer)	180.34		188.51	0–921
DAS28 score	3.80		1.47	1.68–6.7
Anti CCP (titer)	523.95		563.69	0.5–2776
SatO2 (%)	95.62		3.25	80–100
PaO2 (mmHg)	73.26		12.91	47–107
FEV1FVC (%)	76.64		9.92	32–115
FEV1 predicted (%)	85.49		21.71	41–143
FVC predicted (%)	87		22.2	45,4–188
DLCO predicted (%)	65.1		18.51	26.2–108
6MWT distance (meters)	404.69		88.19	199–620
Initial SatO2 6MWT (%)	95.62		2.42	88–99
Final SatO2 6MWT (%)	91.93		3.67	78–98
Acute exacerbations	9	8.5		
Hospital admissions	20	18.9		
Death	18	17		

RA: rheumatoid arthritis; ILD: interstitial lung disease; HRCT: high-resolution computed tomography; UIP: usual interstitial pneumonia; NSIP: non-specific interstitial pneumonia; OP: organizing pneumonia; ESR: erythrocyte sedimentation rate ; CRP: C-reactive protein; RF: rheumatoid factor; DAS: disease activity score, Anti CCP: anti-cyclic citrullinated peptide, FEV1: forced expiratory volume in one second, FVC: forced vital capacity; DLCO: diffusion capacity for carbon monoxide; 6MWT: six minutes walking test.

### Predictors of disease progression

**Figure 1:**
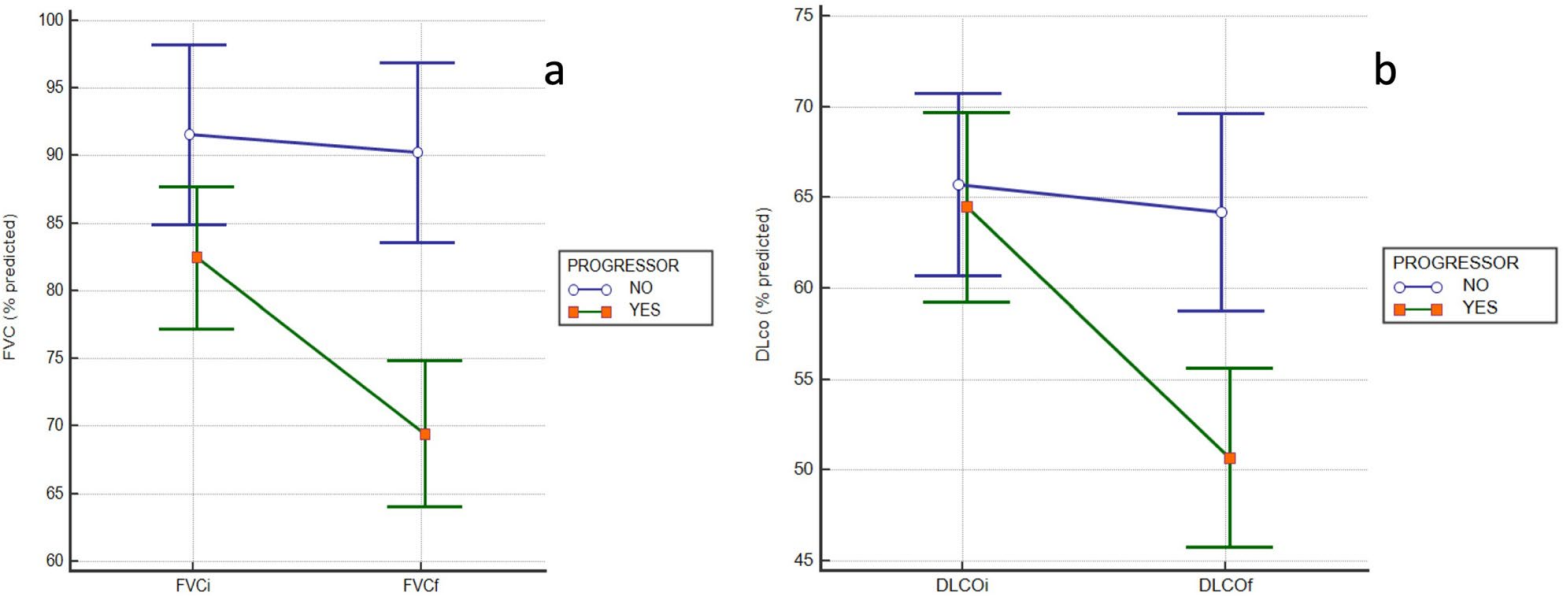
Respiratory functional impairment of the global recruited cases differentiating between groups of progressive and non-progressive patients. FVC (%) impairment is represented in the graph (**a**) and DLCO (%) impairment is represented in the graph (**b**).

After defining the pre-specified variables of ILD progression, 53 patients (50%) presented disease progression. Of those progressors, 35 patients presented a FVC decline > 10%, 25 presented a decrease > 15% of DLCO and 24 an increase of the lung fibrotic signs in the HRCT. The respiratory functional impairment (FVC% and DLco%) is represented differentiating between the groups of progressive and non-progressive patients in the Fig. 1a, b.

**Figure 2:**
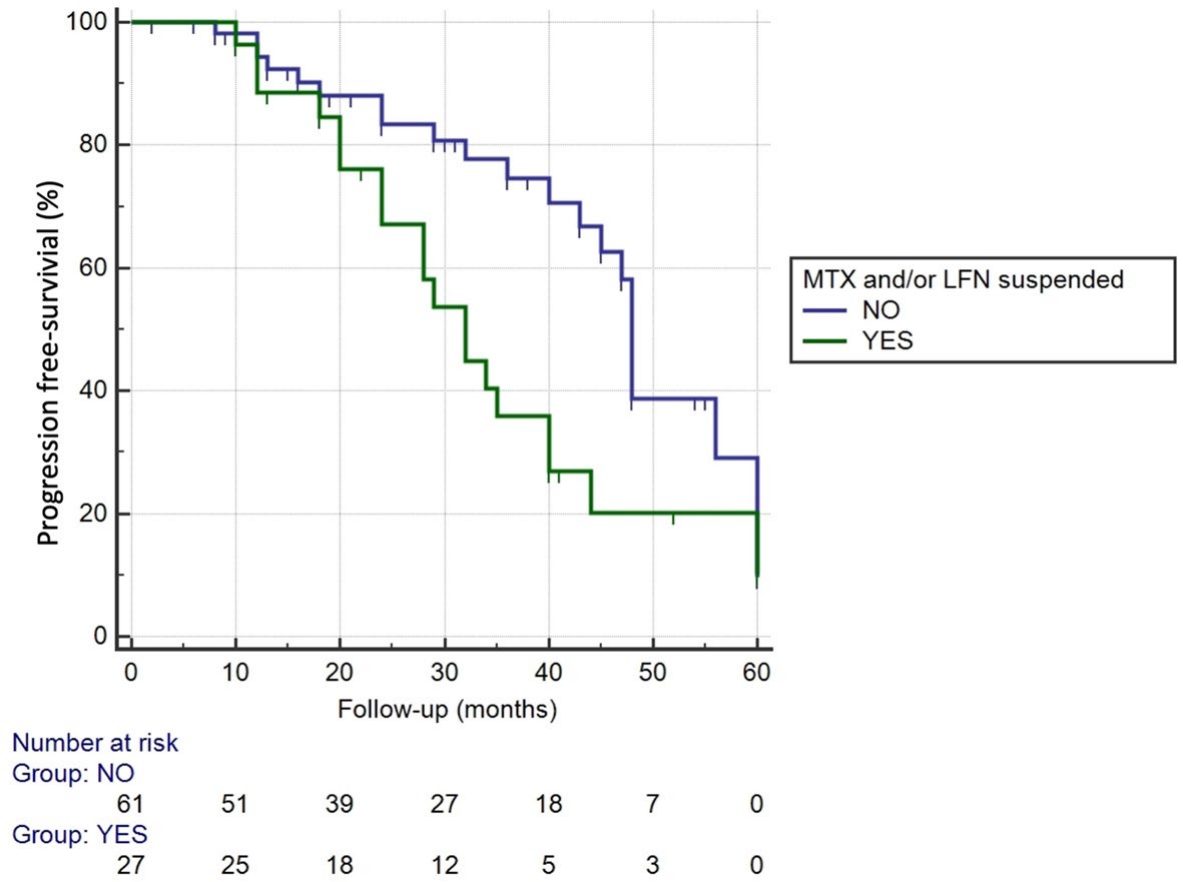
Kaplan–Meier curve for disease progression in RA-ILD during five years period, grouped by discontinuing or not MTX and/or LFN at the moment of ILD diagnosis (Log Rank Test: *p* = 0.0139).

After multivariate COX analysis, withdrawal of MTX or LFN after ILD diagnosis was associated to ILD progression in the first 5 years (HR 2.18, 95% CI 1.14–4.18, *p* = 0.019) (Table 2). From 88 patients that were treated with MTX or LFN previous ILD diagnosis , the medication was changed in 27 cases after the ILD identification due to the potential drug-induced ILD effect of MTX (n = 22) or the existence of extensive pulmonary fibrosis (n = 5). Patients in whom MTX and/or LFN were suspended had initially similar radiological pattern (*p* = 0.874) and respiratory function test values, FVC and DLCO, compared to those who were not discontinued (Supplementary data, Table S1). Disease progression free-survival curves over time are displayed in Fig. 2.

**Table 2:** Cox analysis of progression predictors.

Variable	Univariate		Multivariate	
	HR (95% CI)	*p*	HR (95% CI)	*p*
Age	1.02 (0.98–1.06)	0.2345		
Male, sex	1.31 (0.71–2.41)	0.3884		
Ever smoker	0.97 (0.52–1.81)	0.9331		
Dyspnea 2–3 mMRC	0.81 (0.4–1.66)	0.5826		
Crackles	2.06 (1.01–4.2)	0.0487	1.76 (0.91–3.41)	0.0925
CRP > 3 (mg/L)	0.74 (0.36–1.50)	0.4109		
ESR > 77 (mm/H)	0.74 (0.26–2.12)	0.5831		
RF titer > 70	0.64 (0.29–1.4)	0.2672		
FVC predicted (%)	0.99 (0.97–1.002)	0.1038	0.99 (0.97–1.01)	0,1574
DLCO predicted (%)	0.99 (0.97–1.008)	0.2741		
UIP pattern on HRCT	1.79 (0.96–3.34)	0.0637	1.72 (0.92–3.2)	0.0899
MTX or LFN discontinuation after ILD diagnosis	2.08 (1.12 -3.85)	0.021	2.18 (1.14–4.18)	0.0190

mMRC: modified Medical Research Council; ESR: erythrocyte sedimentation rate ; CRP: C-reactive protein; RF: rheumatoid factor; FVC: forced vital capacity; DLCO: diffusion capacity for carbon monoxide; UIP: usual interstitial pneumonia; HRCT: high-resolution computed tomography; MTX: methotrexate; LFN: leflunomide.

### Survival predictors

**Figure 3:**
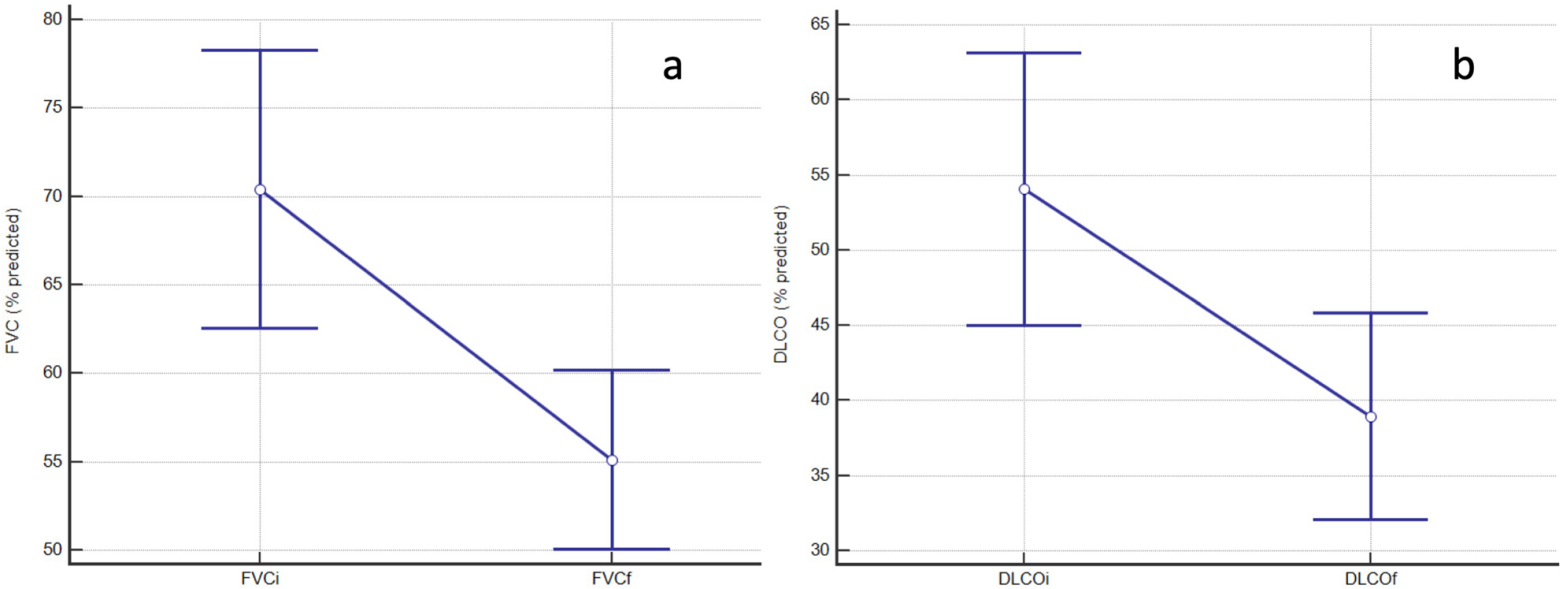
Respiratory functional impairment in deceased patients. FVC (%) impairment is represented in the graph (**a**) and DLCO (%) impairment is represented in the graph (**b**).

Of the 106 subjects reviewed, 18 subjects died (17%). All of them presented fibrotic ILD. Causes of death were mostly related to ILD: 10 patients died of ILD progression, 3 of acute pneumonia and 4 of acute exacerbation of ILD. A missing data about the cause of death was present in one patient and no patient of the cohort underwent lung transplantation. Respiratory functional impairment in deceased patients is represented in Fig. 3a, b.

Patients had a median survival of 41.16 ± 27.94 months. Age (HR 1.33, 95% CI 1.09–1.62, *p* = 0.0045), DLCO predicted (%) (HR 0.85, 95% CI 0.73–0.98, *p* = 0.0246) and final oxygen saturation in the 6MWT (HR 0.62, 95% CI 0.39–0.99, *p* = 0.0465) were independent predictors of mortality in a multivariate model that included all those variables determined in the univariate analysis as a potentially influential predictors (Table 3).

**Table 3:** Univariate and multivariate analysis of mortality predictors.

Variable	Univariate		Multivariate	
	HR (95% CI)	p	HR (95% CI)	p
Male, sex	1.98 (0.77–5.09)	0.1597	0.41 (0.03–4.54)	0.4713
Age	1.10 (1.04–1.17)	0.0013	1.33 (1.09–1.62)	0.0045
Ever-smoker	0.89 (0.35–2.28)	0.8106		
Respiratory symptoms duration until ILD diagnosis	1.03 (1.02–1.05)	0.0001	1.11 (1.01–1.23)	0.035
Dyspnea 2–3 mMRC	1.78 (0.62–5.15)	0.2861		
Crackles	1.02 (0.40–2.59)	0.9612		
UIP pattern on HRCT	2.19 (0.78–6.18)	0.1371	0.45 (0.03–5.89)	0.5450
CRP > 23 (mg/L)	3.61 (1.30–9.98)	0.0136	0.23 (0.01–3.12)	0.2731
ESR > 33 (mm/H)	1.89 (0.66–5.41)	0.2397		
RF titer > 165	0.38 (0.08–1.77)	0.2228		
FVC % pred	0.95 (0.93–0.98)	0.0015	0.99 (0.87–1.13)	0.96
DLCO % pred	0.94 (0.91–0.97)	0.0008	0.85 (0.73–0.98)	0.0246
6MWT distance (meters)	0.99 (0.98–0.99)	0.0026	1.01 (0.98–1.02)	0.8733
6MWT final SatO2	0.83 (0.75–0.92)	0.0002	0.62 (0.39–0.99)	0.0465
GAP index^a^	3.25 (2.04–5.17)	< 0.0001	4.65 (1.59–13.54)	0.0051
CPI index^b^	1.09 (1.04–1.14)	0.0001	1.12 (1.03 -1.22)	0.0092

mMRC: modified Medical Research Council; UIP: usual interstitial pneumonia; HRCT: high-resolution computed tomography; CRP: C-reactive protein; ESR: erythrocyte sedimentation rate ; RF: rheumatoid factor; FVC: forced vital capacity; DLCO: diffusion capacity for carbon monoxide; 6MWT: six minutes walking test; GAP: gender [G], age [A], and lung physiology variables [P] index; CPI: composite physiologic index.

^a^Adjusted by ESR > 33 mm/H, CRP > 23 mg/L, radiological pattern on HRCT, respiratory symptoms duration until ILD diagnosis, and final oxygen saturation in the 6MWT.

^b^Adjusted by ESR > 33 mm/H, CRP > 23 mg/L, radiological pattern on HRCT, respiratory symptoms duration until ILD diagnosis, final oxygen saturation in the 6MWT and age.

**Figure 4:**
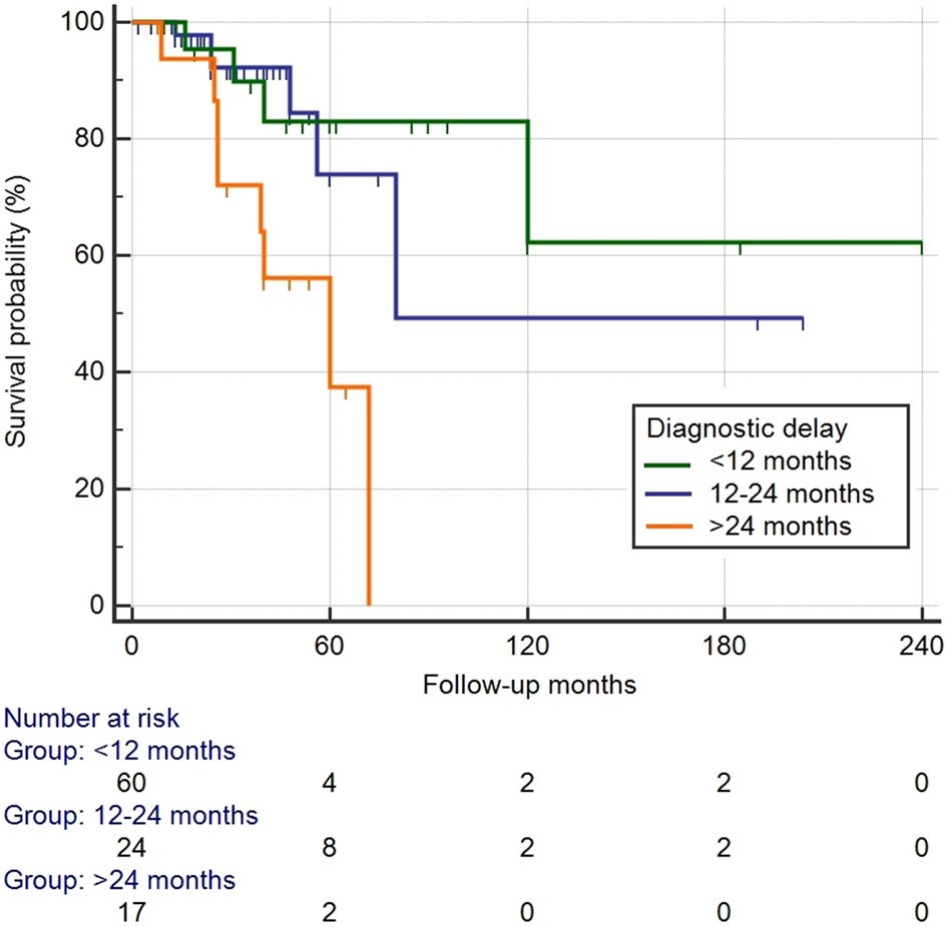
Kaplan–Meier survival curves for patients with RA-ILD. Overall survival according for diagnostic delay (Log Rank Test: *p* = 0.0051).

The median time to ILD diagnosis after appearance of respiratory symptoms in RA patients was 14.36 ± 15.3 months. This information was available for 101 subjects. Also, diagnostic delay was an independent predictor of survival (HR 1.11, CI 1.01–1.23, *p* = 0.035) in the multivariate analysis, with no significant differences depending on received treatments or initial pulmonary functional severity (Supplementary data, Tables S2 and S3). The representation of survival by Kaplan–Meier curves, dividing the diagnostic delay in less than 12 months, 12–24 months and more than 24 months from the beginning of the respiratory symptomatology is displayed in Fig. 4.

Both, CPI and GAP indexes were predictors of mortality in the univariate Cox analysis. After adjusting for radiological pattern on HRCT, RPC > 23 mg/L, ESR > 33 mm/H, ILD diagnostic delay and final oxygen saturation in the 6MWT, GAP index remained as a significant predictor of mortality (HR 4.65, 95% CI 1.59–13.54, *p* = 0.0051). Furthermore, CPI index was a significant predictor of mortality in the multivariate analysis adjusted by age, radiological pattern on HRCT, ESR > 33 mm/H, RPC > 23 mg/L, ILD diagnostic delay and final oxygen saturation in the 6MWT (HR 1.12, 95% CI 1.03–1.22, *p* = 0.0092) (Table 3).

**Figures 5:**
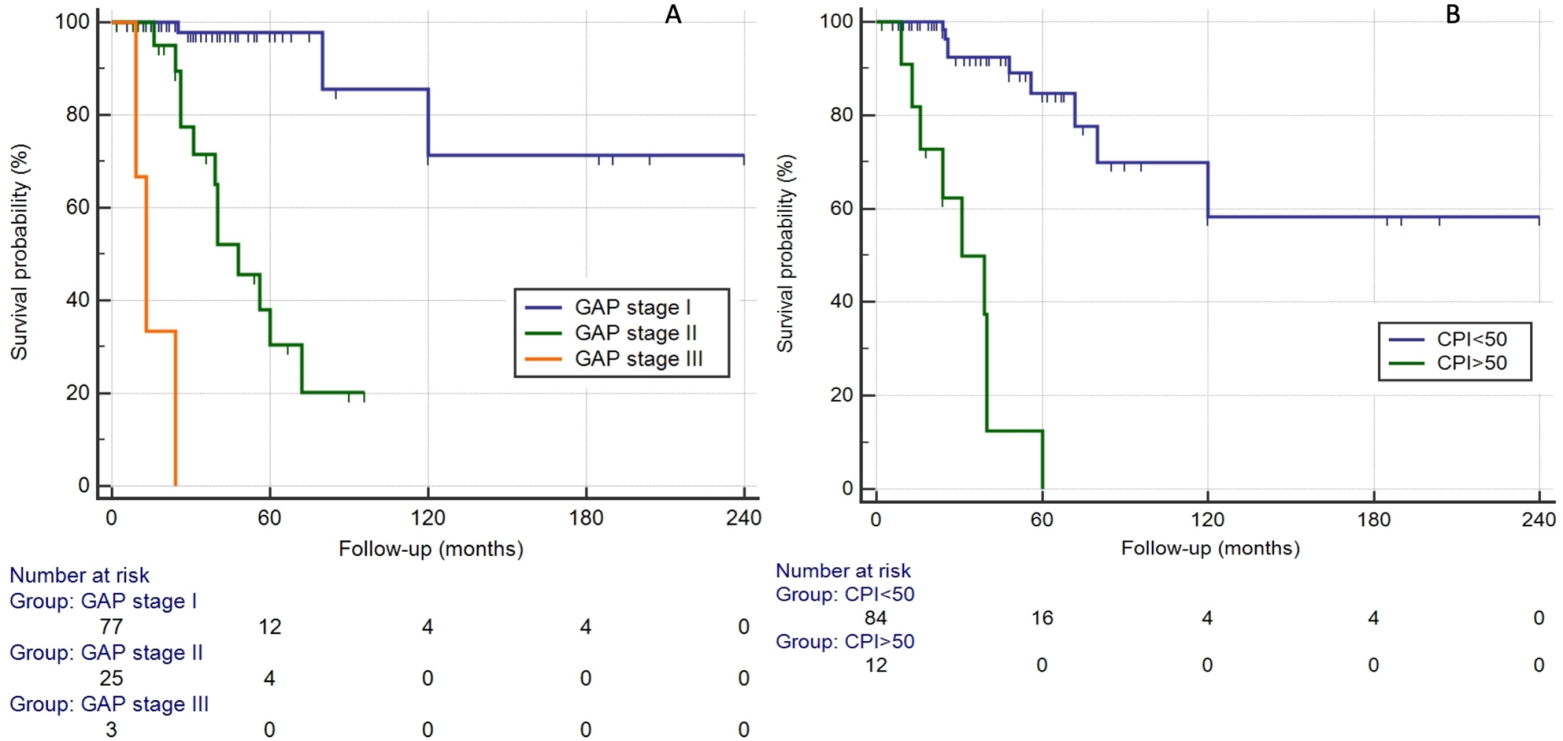
Kaplan–Meier survival curves for patients with RA-ILD. Survival according for GAP stage (A) (Log Rank Test: *p* < 0.001) and for CPI with cut-off point of 50 (B) (Log Rank Test: *p* < 0.0001).

**Figure 6:**
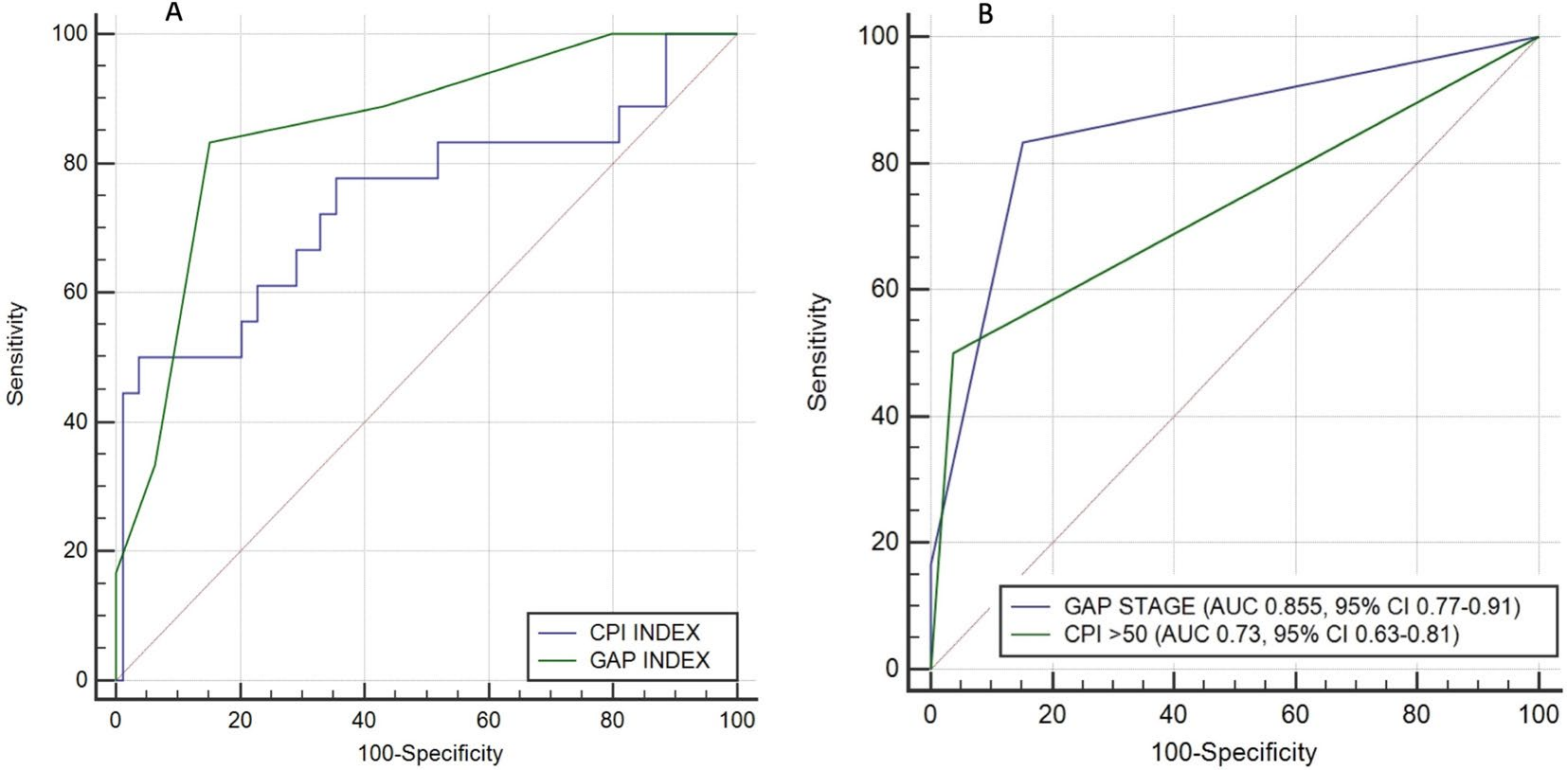
(**A**) ROC curve for CPI and GAP indexes. For CPI, the AUC was 0.742 and the best cut-off point for predicting survival was 50.58 (sensitivity of 50% and specificity of 96.2%). The AUC for GAP ROC curve was 0.857 with a best cut-off point of 3 (sensitivity 83.3% and specificity 85.2%). (**B**) Receiver operator characteristic (ROC) curves for GAP stage (I, II and III) and CPI (with a cut-off point of 50) to predict mortality in AR-ILD patients.

Among the deceased subjects, the median survival was 76.66 ± 46.23 months (n = 3) in the GAP stage I, 38.03 ± 17.35 months (n = 12) in the stage II and only 18 ± 9,54 months (n = 3) in the stage III. The best cut-off point of CPI index for predicting mortality was 50.58. The median survival was 51 ± 34.31 months (n = 9) for patients with CPI < 50 points. Nevertheless if the CPI score was > 50 points, the median survival was 31.33 ± 16.26 months (n = 9) (Fig. 5A, B) . The predictive value of CPI and GAP stages was assessed by ROC curve analysis (Fig. 6A, B).

### Risk of respiratory infection in ILD-RA treatments

Of the 106 RA-ILD subjects, 21 (19.8%) suffered respiratory infectious events. Ten patients (9.4%) were being treated with corticosteroids plus azathioprine and 18 (17%) with biological agents. Logistic regression model, as reflected in Table 4, showed that the association of azathioprine plus corticosteroids was related with an increased risk of respiratory infection (OR 20.86, 95% CI 3.5–124.28, *p* = 0.0008).

**Table 4:** Logistic regression model for respiratory infection risk.

Variable	Univariate		Multivariate	
	OR (95% CI)	*p*	OR (95% CI)	*p*
Corticosteroids plus azathioprine	13.66 (3.15–59.23)	0.0005	20.86 (3.50–124.28)	0.0008
Flu vaccination	1.22 (0.37–4.07)	0.7424	4.28 (0.67–27.29)	0.1241
Pneumococcal vaccination	1.21 (0.46–3.15)	0.6964	0.84 (0.27–2.56)	0.756
Antibiotic prophylaxis	4.37 (0.5781–33.00)	0.153	5.20 (0.50–53.49)	0.1655
Biological treatment	1.73 (0.54–5.55)	0.3561	1.57 (0.38–6.42)	0.53

## Discussion

This is the first study that has identified diagnostic delay as a variable associated with an increased mortality in RA-ILD patients, independently of received treatment. The suspension of MTX and/or LFN was more frequently present among those cases that presented ILD progression. RA-ILD is the most frequent extra-articular manifestation in RA and it's considered the second cause of mortality after cardiovascular comorbidities in RA^4,6^.

The patient characteristics of this cohort are consistent with the previous published data^29,30^, highlighting the high prevalence of patients with tobacco exposure as well as the predominant UIP radiological pattern at diagnosis. Furthermore, in line with previous work^6,31^, age and pulmonary function were identified as predictive mortality variables.

Interestingly, this study demonstrates that the ILD diagnostic delay is a predictive factor of mortality in RA-ILD. After multivariate analysis, disease severity, radiologic pattern, sex and age did not influence in this result. IPF studies had suggested that the diagnostic delay increases mortality, due in part to the late evaluation by a multidisciplinary committee and the potential access to specialized and comprehensive treatment management^32–34^.

Regarding other predictors of mortality, age, DLCO at diagnosis and significant desaturation to effort in 6MWT were independent predictive variables in this cohort. In our study, survival was 41 months on average, similar to IPF^8,9,17,35^. There are several studies that have evaluated predictors of mortality in RA-ILD, including the age^8,9,17,35,36^. The main limitations of these studies have always been the methodology and sample size. Other variables as the disease severity assessed by DLCO have also been confirmed by Zamora et al.^11^. We also suggest desaturation during effort as a prognostic marker in RA-ILD, which is already known as poor prognostic factor in IPF but it had not yet been suggested in RA-ILD population^37^.

No significant differences in mortality were found according to the HRCT pattern of presentation, although all of them showed lung fibrotic features. Previous studies have suggested a different survival in RA-ILD with UIP pattern vs NSIP pattern^38^. However, this has not been a universal finding^15^, with a recent study by Zamora-Legoff et al. proving no association between different patterns and mortality^16^. The discrepancies in the previous reported cohorts could be due to the heterogeneity of NSIP and the broad spectrum of progression depending on the presence of fibrotic changes. Similar to IPF, the clue would be the presence of fibrotic radiological findings that are associated with disease progression; honeycombing or traction bronchiectasis^39^. Therefore, a RA-ILD patient with a UIP or a fibrotic NSIP pattern would have higher probability to death or disease progression than non-fibrotic NSIP or other forms of RA-ILDs.

The existence of this large number of prognostic variables of survival suggests the possibility of using multidimensional models in RA-ILD. Several multidimensional risk scales have been created and validated in ILD including gender, age and lung function (GAP index) for IPF^18^ and subsequently ILD-GAP index for non-IPF ILD^40^. In the Zamora-Legoff study, the GAP model demonstrated good discriminative and predictive value for RA-ILD in contrast to the ILD-GAP model which did not perform well^16^. This partially coincides with our results where the GAP index is a good marker of mortality in the multivariate analysis when adjusted for all those variables that can be predictive and are not included in the index itself. As we have shown, Numri et al. demonstrated in a multivariate analysis adjusted for age that the CPI index was also a predictor of mortality together with DLCO^20^.

MTX and LFN are first-line drugs in the treatment of RA to which other drugs are added during the course of the disease^41^. The association between MTX and acute pulmonary toxicity is well known. Sathi et al. estimated the incidence of MTX-induced pneumonitis at 1 per 192 patient-years and usually occurs during the first year of treatment^42^. Since MTX can exacerbate a preexisting lung disease the American College of Rheumatology recommends not using it in patients with symptomatic RA-ILD^43^. However, the association between MTX and appearance of a chronic ILD other than a hypersensitivity reaction is not so clear. Recent studies do not support this association^44^. On the other hand, LFN has been less frequently associated with ILD exacerbation or development of new ILD^45^. In our cohort, discontinuation of MTX or LFN after the diagnosis of fibrotic ILD in RA patients was associated with disease progression^46^. Our observation, together with recent data, suggests that withdrawing MTX in fibrotic ILD patients doesn’t avoid disease progression and also it points the fact that prospective clinical trials are required for evaluating the exact role of MTX in fibrotic RA-ILD^43^. Thus, we consider that it may be necessary to monitor lung function and perform an HRCT before starting MTX or other drugs that may present pulmonary toxicity.

Regardless of the role of MTX and LFN in RA-ILD disease, we have to consider adding a treatment focused on lung involvement, such as biological agents (e.g. rituximab, abatacept) or antifibrotic treatment such as nintedanib after the recent clinical trials in progressive pulmonary fibrosis. We must individualize the treatment, differentiating the radiological pattern, the severity and progression of lung involvement to better target the proper treatment from a multidisciplinary approach^39–41^.

Respiratory infection or community-acquired pneumonia are the most common type of infections seen in patients with RA^47^. A retrospective study defined serious infection as the requirement of antimicrobial therapy and hospitalization. The authors observed that the highest infection rate was in those patients with a daily prednisone use > 10 mg per day^48^. The use of corticosteroids concurrently with another immunosuppressant such as azathioprine increased mortality, hospitalization rate and serious adverse events in IPF mainly due to pulmonary infection and the presence of telomere shortening^49^. Similar results were obtained in the PANTHER study where increased risks of death and hospitalization were observed in patients with IPF who were treated with a combination of prednisone, azathioprine, and acetylcysteine, as compared with placebo^50^. In our study it seems that the use of prednisone at a dose greater than 10 mg together with azathioprine is associated with a higher occurrence of respiratory infections in a multivariate logistic model corrected by pneumococcal vaccination, flu vaccination and antibiotic prophylaxis. No data on telomere length was available since it is not a current standardized evaluation in these patients.

Our study has several limitations. The major one is related to the retrospective analysis design despite being consecutive cases collected in a multicenter study.

Incomplete clinical information or loss of variables are a constant in this type of studies, hence the need to eliminate some variables such as DAS28 or anti-CCP antibodies to avoid biasing the results in the survival and progression models. Due to the retrospective design of the study and the limited number of patients for analyzing by subgroups (depending on all received therapies), analyzing the impact of other treatments on progression and mortality was not feasible. The majority of patients were assessed in the pulmonology department due to respiratory symptoms. Thus, it is possible that asymptomatic patients and therefore milder cases were not included. Finally, the evaluation of radiological patterns in the HRCT was not centralized, although in all cases it was done in a specialized ILD consultation and after the evaluation in a multidisciplinary discussion with expert ILD radiologists.

In conclusion, the delay in identifying fibrotic ILD in RA patients increases the probability of death or disease progression. The existence of several prognostic variables in RA-ILD makes multidimensional predictive models also useful. Early referral to expert ILD consultation would improve the prognostic and treatment approach, including the proper referral to lung transplant.

## Supplementary Information

Supplementary Information.

## Data Availability

The dataset generated and analysed during the current study is available from the corresponding on reasonable request.

## Abbreviations

6MWT: Six minutes walking test
ACR/EULAR: American College of Rheumatology/European League Against Rheumatism
CCP: Anticyclic citrullinated peptide
CPI: Composite physiologic index
CTD-ILD: Connective tissue disease associated interstitial lung disease
DAS28: Disease activity score 28
DLCO: Diffusion capacity of the lung for carbon monoxide
ESR: Erythrocyte sedimentation rate
FVC: Forced vital capacity
GAP index: Gender, age and physiology variables index
GCPs: Good clinical practices
HRCT: High-resolution computed tomography
IL-6: Interleukin 6
ILD: Interstitial lung disease
IPF: Idiopathic pulmonary fibrosis
KL-6: Krebs von den Lungen 6
LEF: Leflunomide
MTX: Methotrexate
NSIP: Non-specific interstitial pneumonia
RA: Rheumatoid arthritis
RA-ILD: Rheumatoid arthritis associated interstitial lung disease
RPC: Reactive protein C
SatO2: Oxygen saturation
SD: Standard deviation
SEPAR: Respiratory Spanish Society
UIP: Usual interstitial pneumonia
