# Supplementary material for: Diagnostic delay of associated interstitial lung disease increases mortality in rheumatoid arthritis
Source: Sci Rep. 2021 Apr 28;11:9184. doi: 10.1038/s41598-021-88734-2 (PMC8080671; doi:10.1038/s41598-021-88734-2)
Supplement: Supplementary file 1 — Supplementary Information. [file 41598_2021_88734_MOESM1_ESM.docx]

Supplementary data:

| **Table S1. Logistic regression model for relation between MTX and/or LFN withdrawal and pulmonary function severity at ILD diagnosis.** | | | | |
| --- | --- | --- | --- | --- |
| **Variable** | **Univariate** |  | **Multivariate** |  |
|  | **OR (95% CI)** | **p** | **OR (95% CI)** | **p** |
| FVC | 0.98 (0.97- 1.007) | 0.21 | 0.98 (0.96-1.005) | 0.12 |
| DLCO | 1.001 (0.98 to 1.02) | 0.87 | 1.01 (0.98-1.04) | 0.34 |

| **Table S2. Logistic regression model for relation between diagnostic delay and pulmonary function severity after ILD diagnosis.** | | | | |
| --- | --- | --- | --- | --- |
| **Variable** | **Univariate** |  | **Multivariate** |  |
|  | **OR (95% CI)** | **p** | **OR (95% CI)** | **p** |
| FVC | 1.04 (0.98 - 1.1) | 0.13 | 1.08 (0.99 - 1.17) | 0.09 |
| DLCO | 1.0110 (0.96 - 1.06) | 0.66 | 0.95 (0.88 - 1.03) | 0.28 |

| **Table S3. ANOVA analysis for relation between diagnostic delay and treatments after ILD diagnosis.** | | | | |
| --- | --- | --- | --- | --- |
| **Treatment** | **n** | **Mean (months)** | **SD** | **p** |
| No treatment | 12 | 21.41 | 16.96 | 0.14 |
| Steroids | 8 | 9.37 | 11.13 |  |
| Steroids sparing drug | 14 | 7.5 | 9.18 |  |
| Steroids and steroid sparing drug | 49 | 15.06 | 16.97 |  |
| Immunosuppressant treatment and steroids | 1 | 36 | na |  |
| Biologic treatment | 18 | 14.11 | 12.68 |  |
